# Lysine-specific demethylase 1 inhibition enhances autophagy and attenuates early-stage post-spinal cord injury apoptosis

**DOI:** 10.1038/s41420-021-00455-7

**Published:** 2021-04-06

**Authors:** Yang Gu, Dehui Chen, Linquan Zhou, Xin Zhao, Jiemin Lin, Bin Lin, Taotao Lin, Zhi Chen, Zhaohong Chen, Zhenyu Wang, Wenge Liu

**Affiliations:** 1 Department of Orthopedic Surgery, Fujian Medical University Union Hospital, Fuzhou, 350001 Fujian China; 2 School of Health, Fujian Medical University, Fuzhou, 350108 Fujian China; 3 Wound Repair Department, Fujian Medical University Union Hospital, Fuzhou, 350001 Fujian China; 4 Fujian Provincial Key Laboratory of Burn and Trauma, Fuzhou, 350001 Fujian China

**Keywords:** Cell death in the nervous system, Trauma, Cellular neuroscience, Prognostic markers

## Abstract

Neuron death in spinal cords is caused primarily by apoptosis after spinal cord injury (SCI). Autophagy can act as a cellular response to maintain neuron homeostasis that can reduce apoptosis. Although more studies have shown that an epigenetic enzyme called Lysine-specific demethylase 1 (LSD1) can negatively regulate autophagy during cancer research, existing research does not focus on impacts related to LSD1 in nerve injury diseases. This study was designed to determine whether inhibiting LSD1 could enhance autophagy against apoptosis and provide effective neuroprotection in vitro and vivo after SCI. The results showed that LSD1 inhibition treatment significantly reduced spinal cord damage in SCI rat models and was characterized by upregulated autophagy and downregulated apoptosis. Further research demonstrated that using both pharmacological inhibition and gene knockdown could enhance autophagy and reduce apoptosis for in vitro simulation of SCI-caused damage models. Additionally, 3-methyladenine (3-MA) could partially eliminate the effect of autophagy enhancement and apoptosis suppression. These findings demonstrated that LSD1 inhibition could protect against SCI by activating autophagy and hindering apoptosis, suggesting a potential candidate for SCI therapy.

## Introduction

Spinal cord injury (SCI) is a severe neurological disorder that can damage sensation and motor functions and lead to multiple organ dysfunction. Loss of workforce members, the need for long-term treatment, and high rehabilitation costs create a heavy burden on society^1^. Although a range of research efforts has focused on SCI globally, no effective breakthrough exists that can cure SCI. Thus, it is imperative to explore its pathogenesis further and develop effective therapies.

SCI’s pathophysiological progression has two phases. After SCI’s initial phase that is typically caused by mechanical injuries such as acute compression, lacerations, and shear forces, SCI’s second phase causes an ischemic and hypoxic extracellular environment around neurons^2^. The second phase is an available target for therapeutic mediation of SCI, which often exhibits inflammation, hypoxia, glial scar formation, apoptosis, and autophagy^3,4^. Neuronal apoptosis after SCI that results in hypoxic-ischemic damage is a major cause of neurological deficits and mortality^5^. Therefore, it is critical to study the mechanisms of SCI to reduce apoptosis and improve the prognosis of SCI rapidly.

Under stressful extracellular conditions, autophagy is a cellular response to maintain homeostasis of cell structure and function^6^. Autophagy and apoptosis exhibit close interactions, which have been studied extensively in the field of neurotrauma^7,8^. The overall concept involves activating a suitable degree of autophagy in acute SCI cases to allow lysosomes to degrade damaged mitochondria and other malfunctioning components, which limits apoptosis and creates cell structures that promote cell development and proliferation^9^. Also, autophagy receptors can attenuate cell death by selectively decreasing the abundance of proapoptotic signal transducers in the cytosol^10^. Several animal experiments have revealed the extent of decrease in apoptosis in cases of severe spinal cord contusion and compression when autophagic flux was promoted^11,12^. Thus, regulating autophagy with targeted drugs could be a potential method to decrease neuronal apoptosis after SCI.

Autophagic processes are regulated primarily based on movement initiated by nutrient and energy-related sensors, including 5′ AMP-activated protein kinase or mechanistic target of rapamycin kinase complex 1^13^. Moreover, developing outcomes related to epigenetic activities that control the autophagic process could provide different areas of research focus and present ways to create new treatment methods for SCI^14^. Lysine-specific demethylase 1 (LSD1 or KDM1A) is a flavin adenine dinucleotide (FAD)-dependent epigenetic enzyme that is a lysine demethylase acting on H3K4me1/2 and H3K9me1/2 to regulate gene expression^15^. Research in a range of cancer cell lines demonstrated that LSD1 was an important negative regulator for genes that are essential during autophagy and apoptosis^16–18^. Thus, targeting LSD1 was recognized as a potential option for treating cancer, based on studies using a range of LSD1 inhibitors^19^. Although LSD1 has been widely studied in relation to cancer, little is known about its neurological functions. We hypothesized that LSD1 inhibition might function similarly to active autophagy in neurons after SCI. Accordingly, LSD1 inhibitors could be used in SCI to promote autophagy and inhibit apoptosis. This study investigated the impacts of LSD1 inhibition after SCI in vitro and in vivo and explored the relationships among LSD1 and autophagy and apoptosis development, which might provide a potential strategy for treating SCI.

## Results

### LSD1 inhibitor SP2509 protected spinal cord tissue and improved functional recovery from SCI

**Fig. 1:**
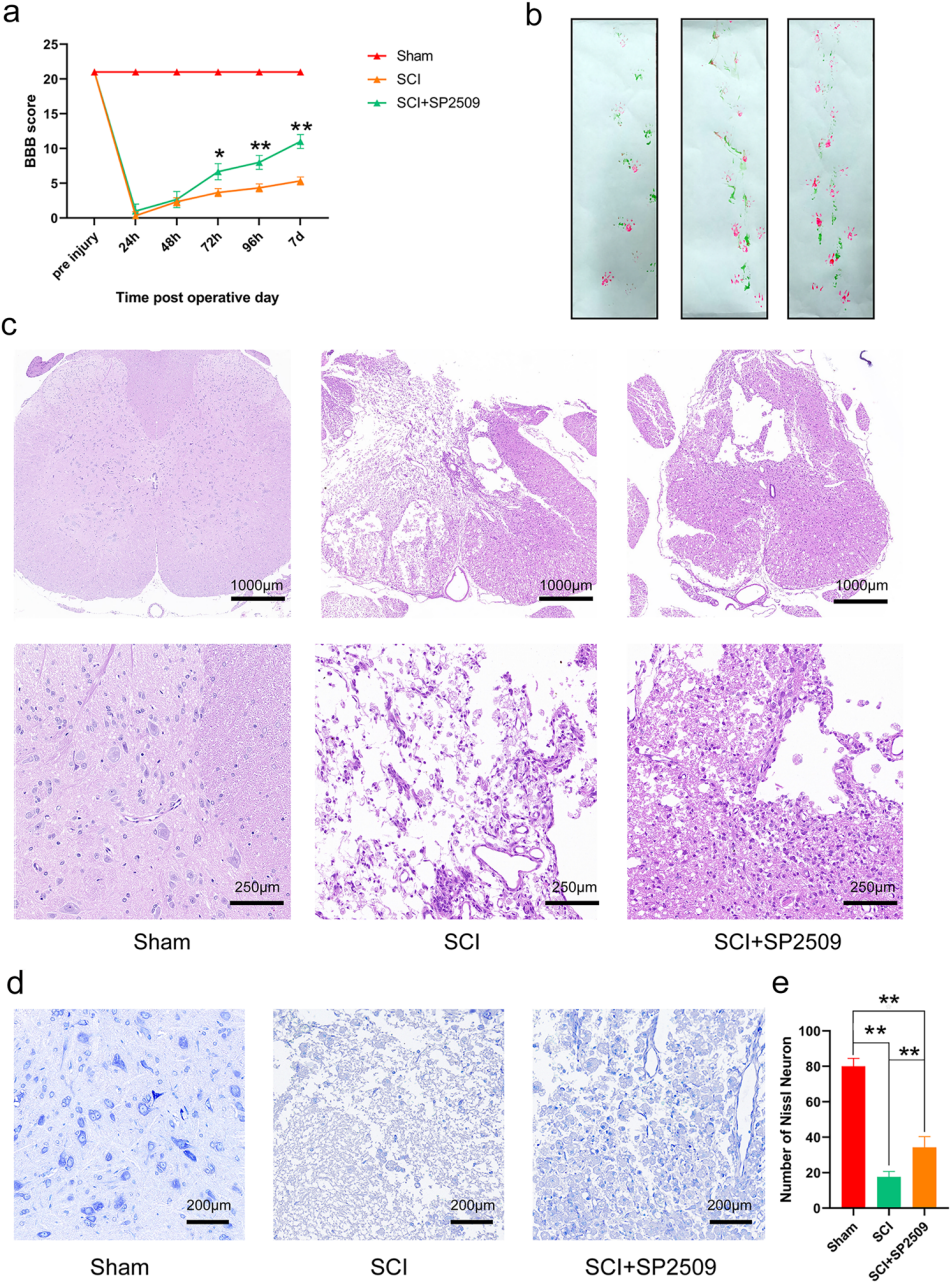
Pharmacologically inhibiting LSD1 based on SP2509 enhanced function recovery and protected spinal cord tissue. **a** BBB scores at various times following spinal cord contusions. **b** Example footprints of rats walking 1 week following SCI. Red: paw print—front paw; green: paw print—hind paw. **c** Representative H&E stained transverse sections. Scale bar = 1000 μm. **d**, **e** Nissl staining for testing survival neurons to indicate each group’s motor neuron amount. Scale bar = 1000 or 250 μm. One-way ANOVA and Tukey’s multiple comparisons test was used to compute *p* values for mean ± SD of three independent experiments. *p < 0.05; **p < 0.01.

SP2509 is a novel, FAD modified, non-monoamine oxidase A and B inhibitor of LSD1/KDM1A^20^. It specifically attenuates the binding of LSD1 to repressor element 1 silencing transcription factor co-repressor (CoREST), allowing increased H3K4 methylation^21^. We carried out Basso–Beatie–Bresnahan (BBB) scoring at 7 days following surgery to explore the therapeutic effect of SP2509 on locomotion recovery in the early stage after SCI. The sham group’s mean BBB score was 21, while those of the SCI and SCI + SP2509-treated groups were 5.0 and 10.8 on average. At 24 and 48 h after injury, no significant differences in BBB scores appeared between the SCI and SP2509 treatment groups. The BBB scores were higher in the SP2509-treated group compared to the untreated group from 72 h to 7 days after injury (*p* < 0.01) (Fig. 1a). Specifically, footprints were analyzed manually to evaluate the gait. Compared with the sham group, all animals’ coordination of their forepaw and hind paw movements decreased significantly. The animals in the SP2509 group showed significant recovery in their gait and motor coordination compared with the SCI group at 1 week after contusion (Fig. 1b).

Hematoxylin and eosin (H&E) staining was used to identify tissue structures. SCI led to structural disorders, including prominent edema, hemorrhage, and tissue loss. One week after SCI, significant spinal cord tissue loss appeared in the SCI group compared to the sham group. However, compared to the SCI group, the tissue loss for the SP2509 group had decreased significantly (Fig. 1c).

To study the function of SP2509 related to neuron loss following SCI, anterior horn motor neurons underwent Nissl staining on day 7 after injury. Neurons in the sham group exhibited an integrative and granular-like morphology. The SCI group displayed a significant loss of large neurons compared with the sham group, whereas SP2509 intervention clearly increased the number of neurons in the anterior horn of the spinal cord (Fig. 1d, e). These results demonstrated that SP2509 treatment to inhibit LSD1 expression at 1 week after SCI could drastically enhance protection of spinal cord tissue and functional recovery. However, its mechanism of action requires additional research.

### LSD1 inhibitor SP2509 enhanced cell autophagy and suppressed cell apoptosis in SCI rat spinal cords

**Fig. 2:**
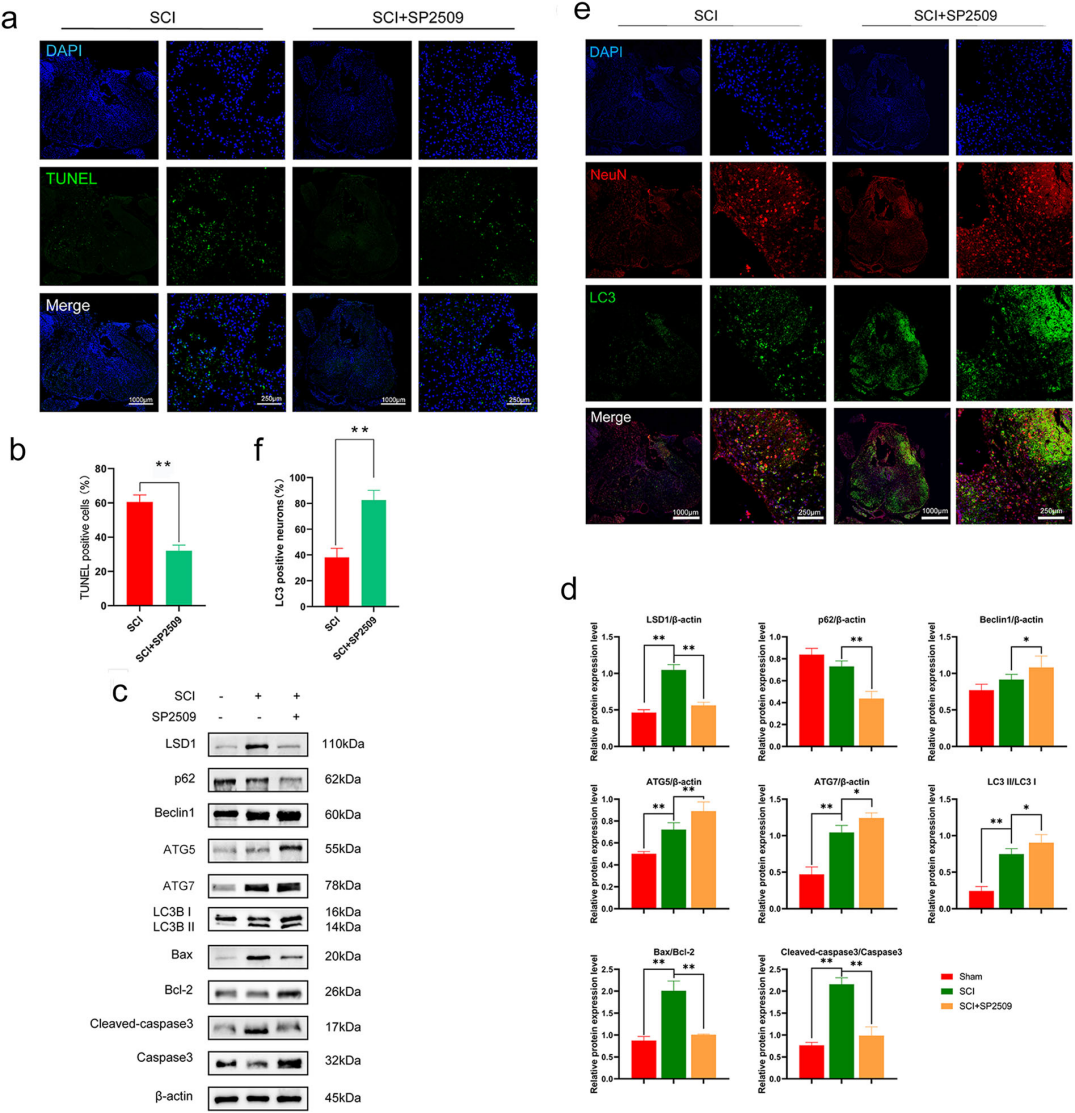
Pharmacologically inhibiting LSD1 based on SP2509 enhanced autophagy and reduces apoptosis in spinal cord tissue. **a** Sample pictures of TUNEL-positive cells from the sham, SCI, and SCI + SP2509 groups at 1 week following injury. Scale bar = 1000 or 250 μm. **b** Counting TUNEL-positive cells in slides. **c** Sample pictures depicting transverse spinal cord sections stained and a quantitative analysis with anti-LC3B and anti-NeuN antibodies in uninjured and injured rats with or without SP2509. DAPI (blue) was used to stain all cell nuclei. Scale bar = 1000 or 250 μm. **d** Analysis of the amount of LC3 positive neurons in traumatic injury area. **e** LSD1 Western blot analysis, autophagy, and apoptosis proteins following injury. **f** Semi-quantitative determination of autophagy and apoptosis protein levels. One-way ANOVA and Tukey’s multiple comparisons test was used to compute *p* values for mean ± SD of three independent experiments. *p < 0.05; **p < 0.01.

To investigate how SP2509 impacted apoptosis, terminal deoxynucleotidyl transferase dUTP nick end labeling (TUNEL) was used to detect DNA fragmentation on day 7 after SCI. TUNEL-positive cells were detected at the injury site in rat spinal cords, and SP2509 treatment dramatically reduced the number of apoptotic cells observed (Fig. 2a, b). Additionally, Western blotting revealed that the expression of LSD1, Bax, and cleaved-caspase3 were upregulated in SCI rats compared to sham rats, and Bcl-2 expression was downregulated. Notably, SP2509 decreased the ratio of Bax to Bcl-2 and for cleaved caspase3 to caspase3 (Fig. 2c, d). Therefore, the LSD1 inhibitor SP2509 promoted neuronal survival by inhibiting apoptosis in spinal cord tissue after SCI.

Autophagy is essential to maintain cell homeostasis and may contribute to decreases in apoptosis. Therefore, we analyzed the effects of SP2509 on the activation of autophagy. LC3 is an autophagic structural protein, and NeuN is a biomarker for neurons. During autophagy, LC3I is combined with phosphatidylethanolamine to form an LC3-phosphatidylethanolaminse conjugate (LC3II), which is tightly bound to autophagosomal membranes^22^. Co-localization of LC3 and NeuN immunofluorescence reveals the degree of autophagy in neurons. Transverse sections of injured spinal cord segments stained with anti-NeuN and anti-LC3B antibodies revealed that SP2509 drastically increased expression of LC3B in injured rat spinal cords (Fig. 2e, f). Furthermore, Western blotting was used to assess the expression of critical autophagy markers, including LC3II/I, Beclin1, p62, ATG5, and ATG7. The SCI group exhibited distinct upregulation of LC3II/I, Beclin1, ATG5, and ATG7, which was accompanied by marked downregulation of p62. Furthermore, compared with the SCI group, SP2509 intervention significantly promoted increased expression of LC3II/I, Beclin1, ATG5, and ATG7, but decreased expression of p62 (Fig. 2c, d). These results demonstrated that SCI could activate autophagy, and using SP2509 to inhibit LSD1 could increase the effect of autophagy.

### Inhibiting LSD1 by SP2509 treatment improved autophagic flux and decreased apoptosis an in vitro model

**Fig. 3:**
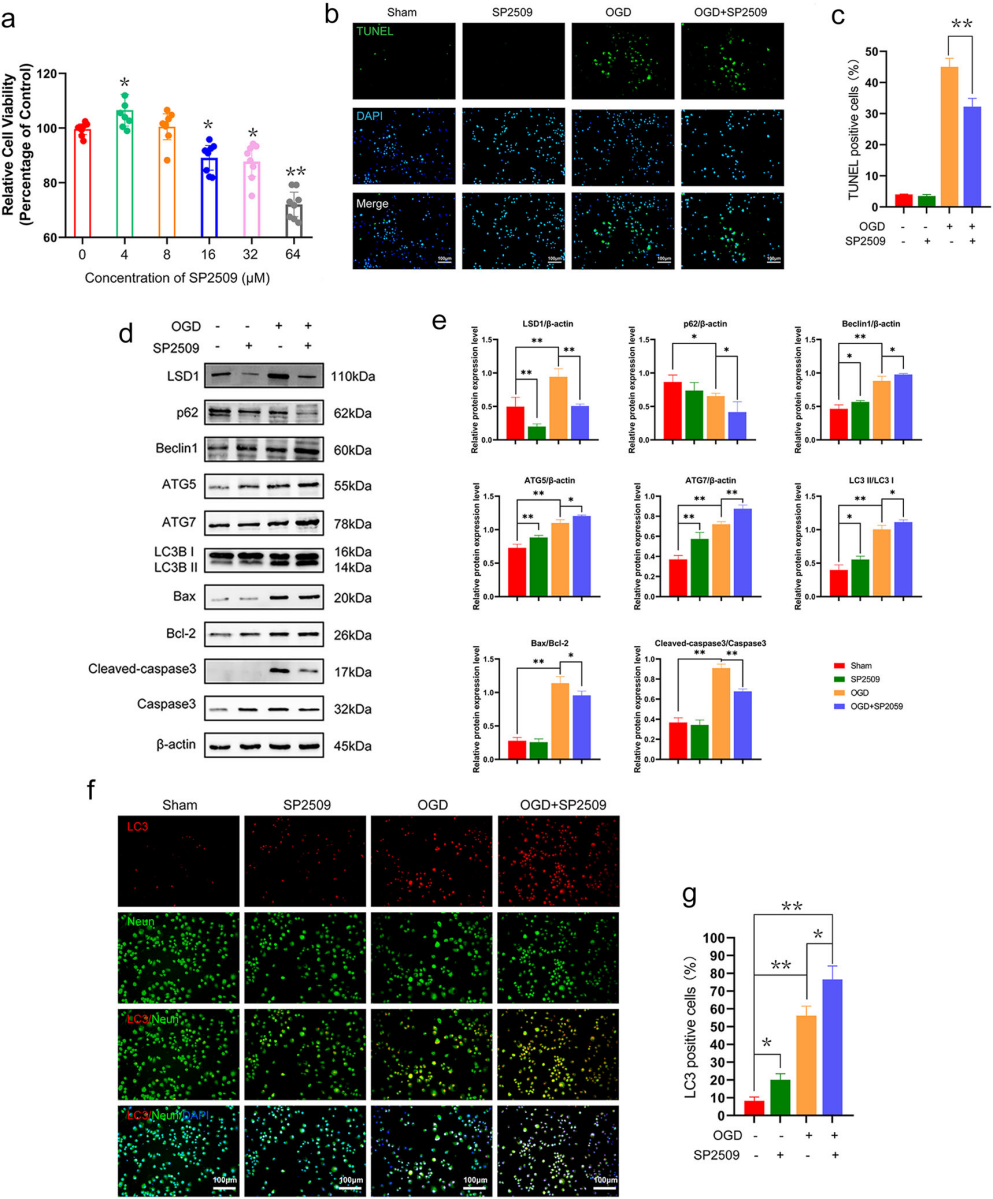
In vitro simulation of PC12 cell damage due to SCI and the therapeutic effect of using SP2509 to pharmacologically inhibit LSD1. **a** CCK-8’s measurement of a quantitative analysis of relative cell viability with various concentrations for treating nocodazole. *n* = 8. **b** PC12 cells apoptosis changes in each group were detected based on TUNEL staining. The SCI + SP2509 group had a lower proportion of TUNEL-positive neurons compared with the SCI group. Scale bar = 100 μm. **c** Counting the TUNEL-positive neurons in the three experimental groups. **d** Western blot analysis of LSD1, autophagy, and apoptosis proteins following injury in different groups. **e** Semi-quantitative detection of autophagy and apoptosis protein levels. **f** Sample images of PC12 cells stained and a quantitative analysis including anti-LC3B and anti-NeuN antibodies in uninjured and injured rats with or without SP2509. DAPI (blue) was used to stain all cell nuclei. Scale bar = 1000 or 250 μm. **g** Analysis of the amount of LC3 positive differentiated PC12 cells. One-way ANOVA and Tukey’s multiple comparisons test was used to compute *p* values for mean ± SD of three independent experiments. *p < 0.05; **p < 0.01.

Oxygen-glucose deprivation (OGD) of neurons was considered a model for the extracellular environment that occurred after SCI in animals. We utilized an in vitro OGD model to mimic SCI damage to investigate how apoptosis and autophagy were related in neurons. Nerve growth factor (NGF) was used to induce differentiation in PC12 cells that were subsequently treated with various concentrations of SP2509 (4, 8, 16, 32, and 64 μM) for the same 2 h of the OGD model induction. Based on the Cell Counting Kit-8 (CCK-8) assay, the cell viability percentage markedly increased with an appropriate dose of SP2509 (4 μM) (Fig. 3a) compared to the control group. Therefore, the concentration of 4 μM SP2509 was chosen for treatment as it significantly enhanced cell viability.

The TUNEL assay results revealed that compared with the OGD group, apoptosis was significantly reduced with exposure to 4 μM SP2509 (Fig. 3b, c). Moreover, Western blotting demonstrated that inhibiting LSD1 by SP2509 decreased the Bax/Bcl-2 ratio, as well as cleaved caspase3/caspase3 ratio in the OGD model (Fig. 3d, e).

Immunofluorescence staining in the OGD model of differentiated PC12 cells revealed a dramatic increase in LC3B (Fig. 3f, g), an autophagic structural protein. Also, following LSD1 suppression, the expression of autophagy markers LC3II/I, Beclin1, ATG5, and ATG7 were upregulated, while p62 was downregulated, indicating enhancement of autophagy flux (Fig. 3d, e). This suggested that drug-based LSD1 inhibition supported the previous results through neuro-autophagy activation in the in vitro OGD model.

### LSD1 knockdown improved autophagic flux and decreased apoptosis in the in vitro model

**Fig. 4:**
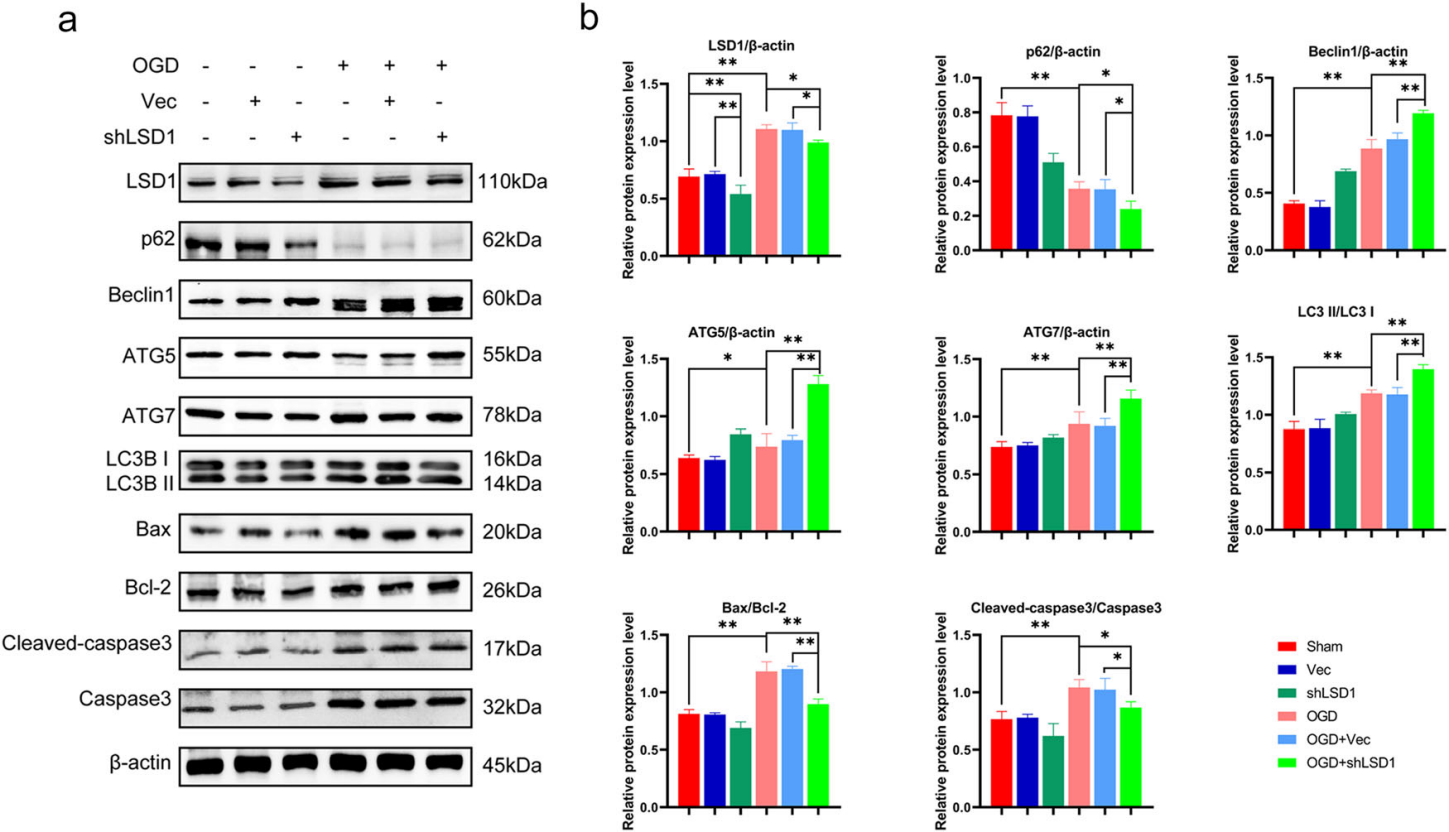
Knockdown of LSD1 upgraded autophagy and downgraded apoptosis related proteins in vitro. **a** LSD1 Western blot analysis, post-injury autophagy, and apoptosis proteins. **b** Semi-quantitative detection of autophagy and apoptosis protein levels. One-way ANOVA and Tukey’s multiple comparisons test was used to compute *p* values for mean ± SD of three independent experiments. *p < 0.05; **p < 0.01.

To further elucidate LSD1’s role in autophagy and apoptosis, we knocked down LSD1 in PC12 cells using lentiviral transduction (shLSD1). Western blots were performed to confirm protein loss. LSD1 protein expression decreased within the shLSD1 cells compared to the sham and empty vector (Vec) groups. Compared with the non-OGD groups, the OGD groups demonstrated an overall increase in LSD1 expression. However, compared to the OGD and OGD + Vec groups, the effect of shLSD1 was maintained in the OGD model. Following LSD1 knockdown in the OGD model, Western blotting also revealed that the levels of Beclin1, ATG5, ATG7, and LC3BII/I markedly increased, while p62 and the Bax/Bcl-2, and cleaved caspase3/caspase3 ratios decreased significantly (Fig. 4a, b).

**Fig. 5:**
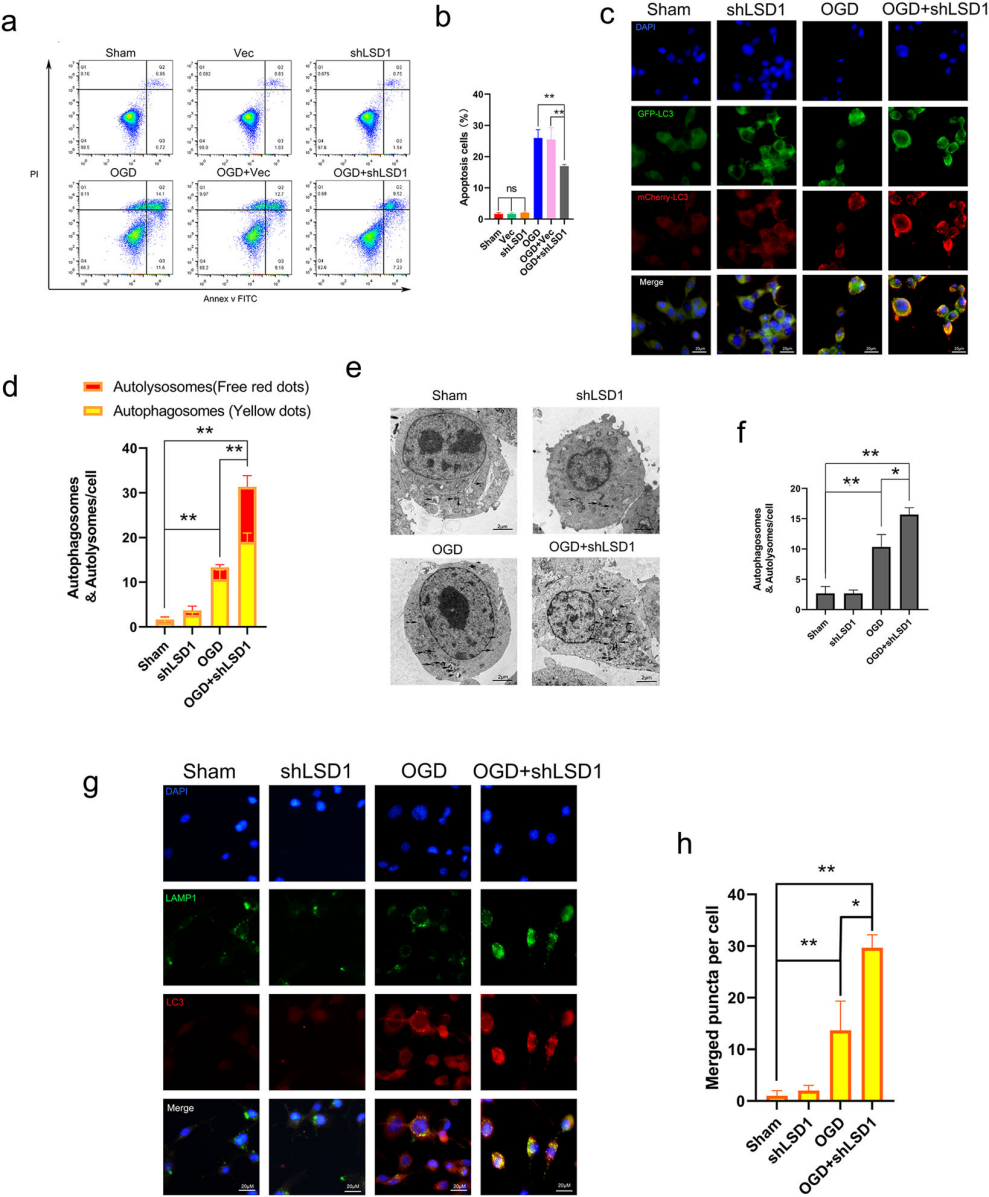
Knockdown of LSD1 upgraded autophagy and downgraded apoptosis in vitro. **a** Annexin V/FITC/PI double staining and flow cytometry detected OGD-induced PC12 apoptosis including or not including LSD1 knockdown. **b** Quantitative results for apoptotic PC12 cells with LSD1 knockdown treatment or lack of treatment. **c**, **d** High magnification fluorescence microscope image of the autophagic flux of mCherry-GFP-LC3 transfected PC12 cells. Autophagosomes were labeled with red and green fluorescence (yellow dots); autophagic lysosomes were labeled with red fluorescence (red dots). The OGD + shLSD1 group had more of these dots compared to the OGD group. Scale bar = 20 μm. **e**, **f** Transmission electron microscopy of autophagosomes of PC12 cells. Sample images indicate usual autophagosomes and autolysomes inside shLSD1-treated OGD PC12 cells. **g**, **h** Immunofluorescent image showing double-labeling with LC3B (an autophagic biomarker, red puncta) and LAMP1 (a lysosomal biomarker, green puncta). The OGD + shLSD1 group had more of merged puncta (yellow puncta) compared to the OGD group. Scale bar = 2 μm. One-way ANOVA and Tukey’s multiple comparisons test was used to compute *p* values for mean ± SD of three independent experiments. *p < 0.05; **p < 0.01.

Annexin V-FITC/PI double staining and flow cytometry revealed that LSD1 knockdown decreased PC12 apoptosis in the OGD model. The shLSD1 + OGD group exhibited fewer apoptotic cells than the OGD only and Vec + OGD groups (Fig. 5a, b). We used a fluorescence microscope and high magnification to observe autophagosomes in cells transiently transfected with adenovirus mCherry-GFP-LC3B, which produced both green (GFP) and red (mCherry) fluorescence. GFP fluorescence was quenched in acidic compartments, whereas mCherry was more stable in a low-pH environment. Consequently, LC3 could be used to ascertain the presence of autophagosomes (GFP-positive and RFP-positive, merged as yellow) and autolysosomes (GFP-negative and mCherry-positive, merged as red). Both yellow and red punctate fluorescence are increased in autophagy activation^23^. The number of autophagosomes and autolysosomes in shLSD1 + OGD increased (Fig. 5c, d). The transmission electron microscope results demonstrated that following OGD induction, some autophagosomes were localized in the PC12 cells. Compared to the OGD only group, shLSD1 + OGD cells contained more autophagosomes (Fig. 5e, f). To further investigate whether shLSD1 + OGD promoted the fusion of autolysosomes, LC3 and LAMP1 (a lysosomal biomarker) were identified using specific antibodies^24^. The extent of co-localization was greater in the shLSD1 + OGD group than the control or OGD only groups (Fig. 5g, h). These results suggested that LSD1 inhibition by LSD1 knockdown enhanced autophagy and apoptosis suppression in the PC12 OGD model, which resembled the results produced by LSD1 inhibition caused by the chemical inhibitor SP2509.

### Administration of 3-methyladenine (3-MA) partially abolished autophagy enhancement and apoptosis suppression in the in vitro model

**Fig. 6:**
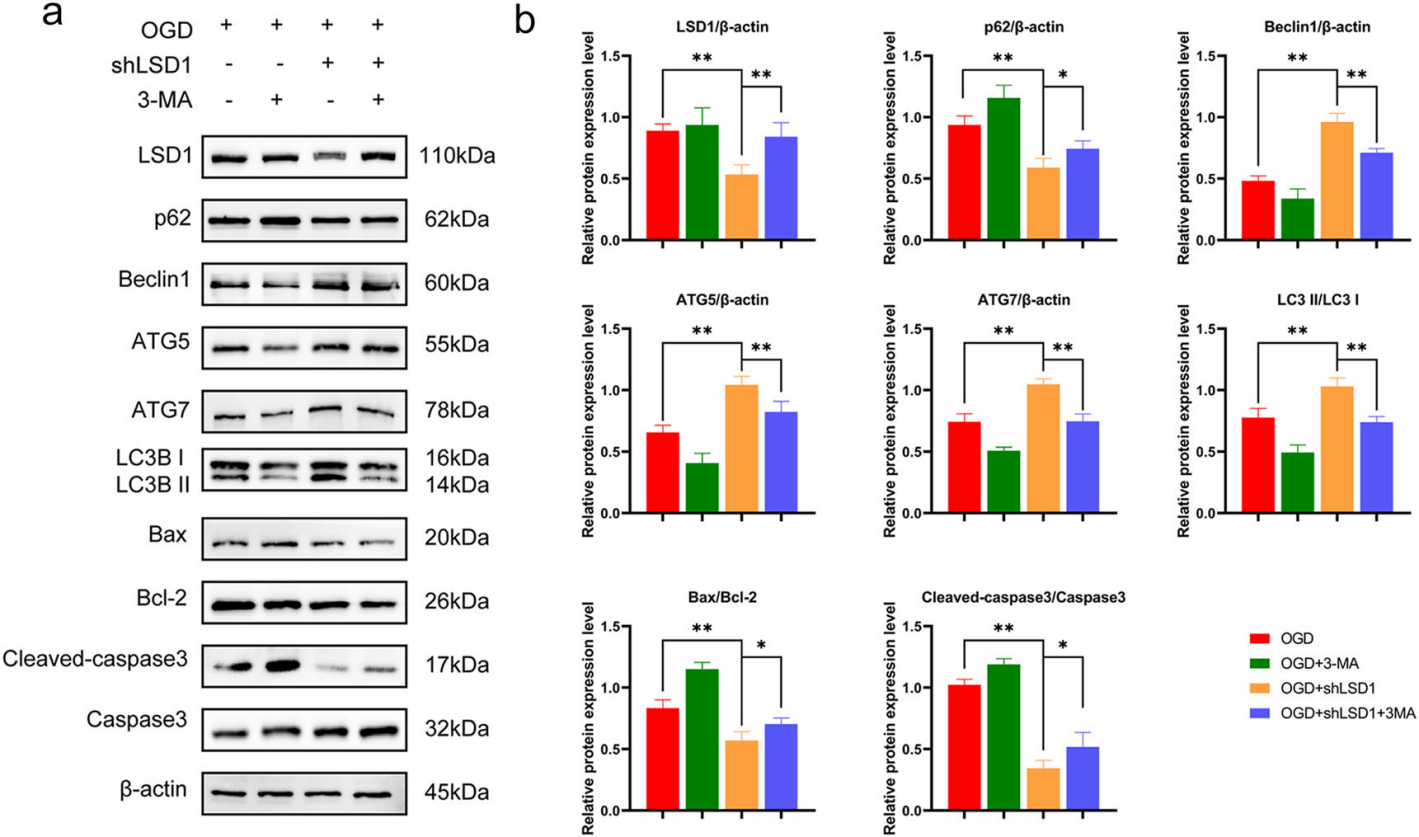
3-MA partially eliminates the effect of autophagy enhancement and apoptosis suppression in vitro. **a** LSD1 Western blot analysis, autophagy and apoptosis proteins following injury in different groups. **b** Semi-quantitative detection of autophagy and apoptosis protein levels. One-way ANOVA and Tukey’s multiple comparisons test was used to compute *p* values for mean ± SD of three independent experiments. *p < 0.05; **p < 0.01.

3-MA is a selective inhibitor of phosphatidylinositol-3-kinase (PI3K) and is well known to suppress autophagy due to its ability to inhibit class III PI3K^25^. 3-MA was applied to determine if LSD1 inhibition treatment after SCI was related to the autophagy effect. Western blotting showed that 3-MA effectively inhibited autophagy in both OGD and OGD + shLSD1 groups, with exceptionally marked inhibition in the OGD + shLSD1 group. Expression of autophagy proteins, including ATG7, LC3II/I, ATG5, and Beclin1, declined significantly, while the expression of p62 and the cleaved caspase3/caspase3 and Bax/Bcl-2 ratios were elevated. However, the expression of ATG5, ATG7, LC3II/I, and Beclin1 were enhanced, and the expression of p62 and the Bax/Bcl-2 and cleaved caspase3/caspase3 ratios were inhibited when the OGD + shLSD1 + 3-MA group was compared with the OGD + 3-MA group (Fig. 6a, b). These results indicated that the therapeutic effect of LSD1 inhibition exhibited a high correlation with enhanced autophagic flux.

## Discussion

Every year, ~10.4–83 SCI cases per million people occur worldwide, and ~2.5 million individuals experience chronic SCI symptoms^26,27^. SCI impairments affect multiple organ systems with devastating effects on patients’ physical, psychological, and social well-being^28^. Spinal cord oxidative stress and neuron apoptosis are the leading causes of the second phase of SCI^29^. Therefore, this study assessed methods that would reduce apoptosis. This study focused on the epigenetic enzyme LSD1, which is related to apoptosis. We found that LSD1 inhibition improved gait and motor coordination as well as the BBB score in rats with SCI, suggesting that it positively impacted spinal cord function after SCI. Also, LSD1 inhibition increased autophagy and decreased apoptosis in rat injured spinal cords in vivo and in the OGD PC12 cell model in vitro. Notably, we found that partly restraining LSD1-related autophagy increased PC12 cells in vitro, indicating that autophagy could regulate apoptosis. These findings further demonstrated that pharmacologically reducing LSD1 could be a potential therapeutic method to treat SCI.

It has been noted that in human tissue and animal models, apoptosis is the primary cause of death for spinal cord neurons^30^. Apoptosis is primarily mediated by the Bcl-2 and caspase families. As a regulator of cell apoptosis and survival, the Bax/Bcl-2/cleaved caspase3 apoptotic signaling pathway is involved in a range of diseases^31,32^. An appropriate Bax (proapoptotic signal) and Bcl-2 (anti-apoptotic molecule) ratio is essential for prolonging the structure and function of mitochrondria^33^. Increased Bax/Bcl-2 and cleaved caspase3/caspase3 ratios might result in caspase-dependent apoptosis^34^. In this study, LSD1 inhibition reduced neuron apoptosis in vivo and in vitro in some cases by acting on the mitochondrial pathway, as shown by augmented expression of Bcl-2 and a gradual decrease in Bax and cleaved caspase3 levels.

Several studies have suggested that autophagy can protect spinal cord neurons against early-stage SCI-induced apoptosis^35–37^. Autophagy is indispensable for normal neuronal homeostasis, and its dysfunction can result in neuron death^38^. LC3 is the most reliable and important indicator of autophagy induction in mammals. During autophagy activation, LC3 can convert from LC3I to LC3II and translocate from the cytosol to autophagosomal membranes^39^. Thus, the LC3II/I ratio is an indicator of autophagic induction. Beclin1 was the first mammalian autophagy protein described as interacting with Bcl-2. When Bcl-2 and Beclin1 dissociate, Beclin1 forms different complexes to induce autophagy^40^. Some primary autophagy regulators are ATG5 and ATG7. ATG7 facilitates the covalent attachment of the protein ATG12 to ATG5, which is based on a conjugation system similar to ubiquitin. ATG5 and ATG12 form a homo-oligomeric complex with ATG16 that is necessary for phagophore elongation during autophagosome formation^41^. Also, p62 can interact with LC3II, and accumulation of p62 signals autophagy inactivation^42^.

Several studies have revealed that LSD1 is an essential regulator of autophagy in multiple diseases, especially cancer. When LSD1 interacts with transcriptional co-repressors, it represses gene transcription by specifically demethylating H3K4me1/2. In contrast, when LSD1 comes in contact with hormone receptors, it specifically demethylates H3K9me1/2, then promotes transcription^14^. In neuroblastoma cells, LSD1 inhibition regulates sestrin2, which is a target gene that induces autophagy^17^. Moreover, research has shown that LSD1 is part of the postprandial epigenetic repression of hepatic autophagy^43^. LSD1 appears to take part in establishing a stable epigenetic state with multiple ATG promoters. However, LSD1 recruitment to these promoters is currently undefined. This study demonstrated that inhibiting LSD1 in SCI and OGD models induced ATG5 and ATG7 expression, as well as enhanced autophagic flux with p62 downregulation, resulting in decreased neuron apoptosis. Moreover, 3-MA acts to stop autophagic flux and somewhat prevents cell recovery that LSD1 inhibition promotes following OGD, suggesting that inhibition of LSD1 enhances autophagy and restrains apoptosis.

This was the first neurotrauma-related study to show that LSD1 inhibition enhanced autophagy and reduced early-stage apoptosis in vitro and in vivo. One limitation of this study was whether LSD1 specifically targeted H3K4me1/2 and H3K9me1/2 to affect the progress of autophagy through direct action on proteins that were modified after translation. Further research is required to explore these additional mechanisms.

## Conclusion

Our study revealed that inhibiting LSD1 ameliorated early-stage apoptosis of SCI neurons by autophagy via Beclin1, ATG5, and ATG7. These results provide a new direction of investigation in which LSD1 could be used to target inhibitors as a therapeutic strategy for SCI.

## Materials and methods

### SCI animal model and experimental groups

Adult Sprague–Dawley rats (220–250 g) were purchased from Fujian Medical University’s Experimental Animal Centre. Animal care and experimental procedure were approved by Fujian Medical University’s Animal Ethics Committee and conducted based on the National Institutes of Health Guide for the Care and Use of Laboratory Animals. All rats were contained in a facility with a constant temperature of 22–24 °C with 45–55% humidity, as well as a 12-h light–dark cycle with available food and water.

The rats’ SCI models were created based on a modified Allen’s method. The rats were intraperitoneally (i.p.) injected with 1% pentobarbital (50 mg/kg) to create sufficient anesthesia and then underwent a laminectomy at T10 to reveal the spinal cord. The rod used weighed 15 g and with a diameter of 2.5 mm (RWD Life Science Corp, China) and was released at 10 cm above the surgery site. The striking force on the spinal cord measured 150 g cm and its contact time was 3 min. The muscles were later realigned and the wound was sutured. In the sham-operated group, the rats underwent an identical procedure except for spinal cord contusion. During the procedure, a heating pad was used to maintain the rats’ body temperature of 37 °C. After operation, the rats were i.p. injected with cefmetazole (100 mg/kg) daily for 3 days. The rats’ bladders were manually emptied two times per day to aid with urination.

### Animals’ administration of SP2509

Working dilutions consisted 20% cremaphor (Sigma, St. Louis, USA), 20% dimethyl sulfoxide (Sigma, St. Louis, USA) and 60% phosphate-buffered saline (PBS). SP2509 was obtained from Med Chem Express (New Jersey, USA). Stock solutions were prepared by dissolving the components in the working dilutions with a concentration of 5 mg/ml.

Rats were separated randomly in three groups (*n* = 10 for each group), including sham-operated, SCI-only, and Add-SP2509 groups: one was treated with 25 mg/kg SP2509 immediately and 48 h after the wound was closed, while the other two groups were treated with 5 ml/kg working dilutions^20,44^.

### Assessment of locomotor capacity

The exercise capacity assessment used the 21-point (0–21) BBB scale^45^ to assess the rats’ behaviors before and 24, 48, 72, 96 h, and 7 days by two blinded investigators following surgery. Assessments were based on observations of the rats’ unrestricted hindlimb movement in a field at a certain time every morning of the testing period, during which rats were able to walk without restrictions on a field for 5 min. In addition, BBB score was performed immediately after the rats regained consciousness Rats with scores higher than 5 were excluded because they were failed in modeling, and backup rats were used for modeling supplement.

Seven days following surgery, an assessment of the rats’ gait and motor coordination was conducted. Their paws were covered with differently colored dye. They were put on permeable paper in a cage and encouraged to walk directly forward. Their footprint pattern was digitized and an overall image was applied to determine coordination.

### Hematoxylin and eosin, and Nissl staining

The rats underwent anesthetization and transcardially perfused with 0.9% NaCl at 7 days after SCI. Spinal cord tissues of the lesion were carefully obtained, fixed in 4% paraformaldehyde (PFA) for 24 h, and embedded in paraffin for a cross-section. Cross-sections (4-μm thick) were made on slides with poly-L-lysine coating for an analysis of diseased tissue based on H&E and Nissl staining after deparaffinized and rehydrated. A light microscope (Leica, Heidelberg, Germany) was used to capture images. ImageJ software (NIH, Bethesda, MD) was used to determine the numbers of surviving neurons.

### Cell culture, differentiate, and oxygen-glucose deprivation (OGD) model and treatment

PC12 cells have previously been applied in SCI studies in vitro. These come from a rat adrenal pheochromocytoma cell line that resembles a neuron^46–48^. They were purchased from the Cell Bank at the Chinese Academy of Sciences (Shanghai, China) and cultured at the Roswell Park Memorial Institute 1640 medium (Gibco, CA, USA) with 10% equine serum (Gibco, CA, USA, New Zealand origin), 5% fetal bovine serum (FBS; Gibco, CA, USA, Brazil origin), and 1% penicillin/streptomycin (Gibco, CA, USA) at 37 °C in a humidified room with 5% CO_2_.

The PC12 cells were differentiated by being seeded on poly-L-lysine-coated 6-cm plates at 1 × 10^6^ cells/well and cultured for 24 h. RPMI-1640 containing 10% FBS, 50 ng/ml NGF (Sangon Biotech, Shanghai, China) and 1% penicillin/streptomycin then replaced the culture medium. One time per 2 days, the medium was switched until the neurite length was greater than the cell body of the individual cells at the 5th day.

The OGD models were led to induce the death and apoptosis of NGF-differentiated PC12 cells to imitate an ischemic SCI in vivo^49^. PBS was used to wash these cells, which were then incubated in Hanks’ balanced salt solution (Gibco, CA, USA) without serum using a Forma Series II Water Jacketed CO_2_ Incubator (Thermo, PA, USA) with 1% O_2_, 5% CO_2_, and 95% N_2_ for 2 h to stimulate early-stage cell injury.

3-MA (Med Chem Express, New Jersey, USA) with a concentration of 3 mM as in a previous study^50^ was administered during OGD modeling culture for the same time (2 h) to suppress autophagy-related pathway proteins and thereby control autophagosome formation to suppress autophagy.

### Cell viability assay

After adding different SP2509 concentrations, CCK-8 assay was applied to determine PC12 cell viability. PC12 cells were briefly seeded onto 96-well plates (1.5 × 10^4^ per well) with 100 μl of complete medium at 37 °C for 24 h. Next, 10 μl of CCK-8 solution (Dojindo Laboratories, Kyushu, Japan) was put into every well, and the plate underwent 2 h of incubation. Lastly, a microplate reader (BioTek, VT, USA) was used to measure absorbance at 450 and 630 nm.

### Knockdown of LSD1 in PC12 cells

To knockdown the expression of LSD1 protein, a LSD1 shRNA was developed to silence the LSD1 genes. LSD1 shRNA oligonucleotides with the LV2N (U6/Puro) vector was used to construct the Lenti-LSD1 shRNA vectors. The LSD1 shRNA sequences were: forward, 5′-TGGACTTCAAGACGACAGTTCTTTCAAGAGAAG

AACTGTCGTCTTGAAGTCCTTTTTTC-3′, and reverse, 5′-TCGAGAAAAAAGG

ACTTCAAGACGACAGTTCTTCTCTTGAAAGAACTGTCGTCTTGAAGTCCA-3′. Lentiviruses (Lenti-LSD1 shRNA or Lenti-shRNA) (MOI = 20) were applied for transfecting PC12 cells with 5 μg/ml polybrene for 24 h based on the manufacturer’s directions (GenePharma, Shanghai, China). After 4 μg/ml puromycin selection, the PC12 cells were harvested for western blot to evaluate LSD1 knockdown efficiency.

### Double-labeled adenovirus mCherry-GFP-LC3B transfection and autophagy detection

PC12 cells were prepared based on the description and seeded on confocal dishes for 24 h. Based on the manufacturer’s direction, these cells were then differentiated using NGF mentioned above and transfected with mCherry-GFP-LC3B lentivirus (MOI = 20) (Beyotime Biotech, Hangzhou, China) for 24 h. The cells were separated based on four groups: Sham, shLSD1, OGD, OGD + shLSD1. After treatment, they were washed using PBS, fixed in 4% PFA, and observed with an inverted fluorescence microscope (Leica, Heidelberg, Germany). The yellow spots representing autophagic bodies and red spots representing autophagic lysosomes were counted.

### Transmission electron microscope (TEM) assessment autophagy

Following treatment, trypsin was used to detach adherent differentiated PC12 cells, which were then centrifuged. The cell pellet was fixed with a pre-cooled 2% glutaraldehyde solution at 4 °C for 2 h, stained with 2% uranyl acetate solution for 2 h, and dehydrated in an acetone gradient of 50, 70, 90, and 100%. The cells were eventually embedded and ultrathin sections were prepared for analysis using an electron microscope (Hitachi, Tokyo, Japan).

### Cell apoptosis measurements using Annexin V/FITC/PI double staining and flow cytometry

After the treatment, centrifugation at 1200 rpm for 3 min was used to harvest the cells, which were washed two times with PBS. The harvested cells were resuspended in FITC-labeled Annexin V (5 μl; BD Bioscience, CA, USA) as well as PI (5 μl; BD bioscience, CA, USA) in darkness for 5 min, and washed three times with PBS. Flow cytometry (FACSCalibur; BD bioscience, CA, USA) was then used to estimate the cell apoptosis rate.

### Terminal deoxynucleotidyl transferase dTUP nick end labeling (TUNEL) staining spinal cord slices and PC12 cells

Sections of spinal cords along with adherent PC12 cells were fixed, blocked, and incubated with a TUNEL reaction mixture (Roche, Basel, Switzerland) at 37 °C for 1 h. DAPI was used to counterstain the nuclei. Each group’s proportion of TUNEL-positive cells was counted under a fluorescence microscope (Leica, Heidelberg, Germany).

### Immunofluorescence staining of spinal cord slices and PC12 cells

Sections of spinal cords and fixed PC12 cells were made permeable with 0.2% Triton X-100 (Solarbio, Beijing, China) and blocked by diluted 5% bovine serum albumin (Sigma, Shanghai, China) and (0.3% w/v) for 30 min. These were stained overnight at 4 °C with primary antibodies against these proteins: LC3B (1:50, Abcam, Cambridgeshire, England), NeuN (1:100, Abcam, Cambridgeshire, England), and LAMP1 (1:50, Abcam, Cambridgeshire, England). Sections and PC12 cells were washed three times in PBS, and incubated with Cy3- or FITC-conjugated secondary antibodies (1:500, Beyotime Biotech, Hangzhou, China) for 2 h at room temperature. DAPI was then used for 15 min to counterstain nuclei, and a fluorescence microscope (Leica, Heidelberg, Germany) was used to acquire fluorescent images. The same exposure time and conditions were used to acquire all images in order to compare different groups. ImageJ software (NIH, Bethesda, MD) was used for quantification.

### Western blot analysis

Total protein was extracted from cells and tissues, and a BCA assay kit (Boster Biotech, Wuhan, China) was used to measure the protein concentration. Sodium dodcyl sulfate-polyacrylamide gel electrophoresis was used to separate proteins, which were transferred onto polyvinlidene difluoride membranes. Membranes were blocked with 5% slim milk powder for 2 h at room temperature and incubated with antibodies against LSD1/KDM1A (1:4000 Abcam, Cambridgeshire, England), p62 (1:1000, Cell Signal Technology, MA, USA), Beclin1 (1:1000, Cell Signal Technology, MA, USA), ATG5 (1:1000, Cell Signal Technology, MA, USA), ATG7 (1:1000 Abcam, Cambridgeshire, England), LC3B (1:1000 Abcam, Cambridgeshire, England), Bax (1:1000, Cell Signal Technology, MA, USA), Bcl-2 (1:1000 Abcam, Cambridgeshire, England), cleaved-caspase3 (1:500 Abcam, Cambridgeshire, England), caspase3 (1:5000 Abcam, Cambridgeshire, England), and β-actin (as a gel-loading control, 1:1000, Cell Signal Technology, MA, USA) for 12 h at 4 °C. HRP-conjugated secondary antibodies (1:5000, Dingguo, Beijing, China) were used to incubate membranes for 2 h at room temperature, after which an enhanced chemiluminescence reagent (Beyotime Biotech, Hangzhou, China) was used to visualize the immunolabeled bands. ImageJ software (NIH, Bethesda, MD) was used to determine protein expression levels based on densitometry.

### Statistical analysis

SPSS software (Version 25.0, USA) and Prism v8.0 software (GraphPad, CA, USA) was used to analyze all data presented as mean ± standard deviation from at least three independent experiments unless the figure legends specifically stated otherwise. One-way analysis of variance test was used to assess the differences among multiple groups, followed by Tukey’s multiple comparison test. Data are expressed as means ± SEM. Statistical significance was denoted as follows: **p < 0.01, *p < 0.05.

## Data Availability

The datasets used and/or analyzed during the current study are available from the corresponding author on reasonable request.

## References

[1] Kang Y (2018). Epidemiology of worldwide spinal cord injury: a literature review. J. Neurorestoratol..

[2] Shende P, Subedi M (2017). Pathophysiology, mechanisms and applications of mesenchymal stem cells for the treatment of spinal cord injury. Biomed. Pharmacother..

[3] Orr MB, Gensel JC (2018). Spinal cord injury scarring and inflammation: therapies targeting glial and inflammatory responses. Neurotherapeutics.

[4] Luo C, Tao L (2020). The function and mechanisms of autophagy in spinal cord injury. Adv. Exp. Med. Biol..

[5] Wu J, Lipinski MM (2019). Autophagy in neurotrauma: good, bad, or dysregulated. Cells.

[6] Shi J (2018). Wild-type p53-modulated autophagy and autophagic fibroblast apoptosis inhibit hypertrophic scar formation. Lab. Invest..

[7] Fimia GM, Piacentini M (2010). Regulation of autophagy in mammals and its interplay with apoptosis. Cell Mol. Life Sci..

[8] Rong Y (2019). Neural stem cell-derived small extracellular vesicles attenuate apoptosis and neuroinflammation after traumatic spinal cord injury by activating autophagy. Cell Death Dis..

[9] Ray SK (2020). Modulation of autophagy for neuroprotection and functional recovery in traumatic spinal cord injury. Neural Regeneration Res..

[10] Mariño G, Niso-Santano M, Baehrecke EH, Kroemer G (2014). Self-consumption: the interplay of autophagy and apoptosis. Nat. Rev. Mol. Cell Biol..

[11] Liu S (2015). Disrupted autophagy after spinal cord injury is associated with ER stress and neuronal cell death. Cell Death Dis..

[12] Zhang D (2017). Metformin improves functional recovery after spinal cord injury via autophagy flux stimulation. Mol. Neurobiol..

[13] Kim J, Kundu M, Viollet B, Guan KL (2011). AMPK and mTOR regulate autophagy through direct phosphorylation of Ulk1. Nat. Cell Biol..

[14] Ambrosio S, Ballabio A, Majello B (2019). Histone methyl-transferases and demethylases in the autophagy regulatory network: the emerging role of KDM1A/LSD1 demethylase. Autophagy.

[15] Gu FY (2020). Biological roles of LSD1 beyond its demethylase activity. Cell Mol. Life Sci..

[16] Feng SJ, Ye J, Cui MJ, Zheng JH (2016). Lysine-specific demethylase 1 (LSD1) inhibitor S2101 induces autophagy via the AKT/mTOR pathway in SKOV3 ovarian cancer cells. Med. Sci. Monit..

[17] Ambrosio S, Amente CD, Paladino S, Lania L, Majello B (2017). Lysine-specific demethylase LSD1 regulates autophagy in neuroblastoma through SESN2-dependent pathway. Oncogene.

[18] Wang Z (2017). Inhibition of H3K4 demethylation induces autophagy in cancer cell lines. Biochim. Biophys. Acta Mol. Cell Res..

[19] Fang Y., et al. Natural products as LSD1 inhibitors for cancer therapy. *Acta Pharm. Sin. B*10.1016/j.apsb.2020.06.007 (2020). 10.1016/j.apsb.2020.06.007 PMC7305746 32837872

[20] Fiskus W (2014). Highly effective combination of LSD1 (KDM1A) antagonist and pan-histone deacetylase inhibitor against human AML cells. Leukemia.

[21] Fiskus W (2012). Pre-clinical efficacy of combined therapy with LSD1 antagonist SP-2509 and pan-histone deacetylase inhibitor against AML blast progenitor cells. Blood.

[22] Tarique I (2019). LIPOPHAGY: a novel form of steroidogenic activity within the LEYDIG cell during the reproductive cycle of turtle. Reprod. Biol. Endocrinol..

[23] Chung SJ (2017). ADIPOQ/adiponectin induces cytotoxic autophagy in breast cancer cells through STK11/LKB1-mediated activation of the AMPK-ULK1 axis. Autophagy.

[24] Yang R (2019). HIF-1α/PDK4/autophagy pathway protects against advanced glycation end-products induced vascular smooth muscle cell calcification. Biochem. Biophys. Res. Commun..

[25] Bo Q (2020). 3-Methyladenine alleviates experimental subretinal fibrosis by inhibiting macrophages and M2 polarization through the PI3K/Akt pathway. J. Ocul. Pharm. Ther..

[26] Karsy M, Hawryluk G (2019). Modern medical management of spinal cord injury. Curr. Neurol. Neurosci. Rep..

[27] Kumar R (2018). Traumatic spinal injury: global epidemiology and worldwide volume. World Neurosurg..

[28] Ong B, Wilson J, Henzel MK (2020). Management of the patient with chronic spinal cord injury. Med Clin. North Am..

[29] Xia P, Gao X, Duan L, Zhang W, Sun YF (2018). Mulberrin (Mul) reduces spinal cord injury (SCI)-induced apoptosis, inflammation and oxidative stress in rats via miroRNA-337 by targeting Nrf-2. Biomed. Pharmacother..

[30] Bahney J, von Bartheld CS (2018). The cellular composition and glia-neuron ratio in the spinal cord of a human and a nonhuman primate: comparison with other species and brain regions. Anat. Rec. (Hoboken).

[31] Czabotar PE, Lessene G, Strasser A, Adams JM (2014). Control of apoptosis by the BCL-2 protein family: implications for physiology and therapy. Nat. Rev. Mol. Cell Biol..

[32] Khalilzadeh B (2018). Advances in nanomaterial based optical biosensing and bioimaging of apoptosis via caspase-3 activity: a review. Mikrochim. Acta.

[33] Li HT (2018). The effects of icariin on enhancing motor recovery through attenuating pro-inflammatory factors and oxidative stress via mitochondrial apoptotic pathway in the mice model of spinal cord injury. Front. Physiol..

[34] Moldoveanu T, Follis AV, Kriwacki RW, Green DR (2014). Many players in BCL-2 family affairs. Trends Biochem. Sci..

[35] Wang ZY, Lin JH, Muharram A, Liu WG (2014). Beclin-1-mediated autophagy protects spinal cord neurons against mechanical injury-induced apoptosis. Apoptosis.

[36] Wang ZY (2017). Autophagy protects against PI3K/Akt/mTOR-mediated apoptosis of spinal cord neurons after mechanical injury. Neurosci. Lett..

[37] Chen G, Li JD, Wang ZY, Liu WG (2020). Ezetimibe protects against spinal cord injury by regulating autophagy and apoptosis through inactivation of PI3K/AKT/mTOR signaling. Am. J. Transl. Res.

[38] Zhang D, Wang F, Zhai X, Li XH, He XJ (2018). Lithium promotes recovery of neurological function after spinal cord injury by inducing autophagy. Neural Regen. Res..

[39] Aparicio IM, Muñoz PM, Salido GM, Peña FJ, Tapia JA (2016). The autophagy-related protein LC3 is processed in stallion spermatozoa during short-and long-term storage and the related stressful conditions. Animal.

[40] Xu HD, Qin ZH (2019). Beclin 1, Bcl-2 and autophagy. Adv. Exp. Med. Biol..

[41] Wang X (2020). m6A mRNA methylation controls autophagy and adipogenesis by targeting Atg5 and Atg7. Autophagy.

[42] Tanabe F (2011). Accumulation of p62 in degenerated spinal cord under chronic mechanical compression: functional analysis of p62 and autophagy in hypoxic neuronal cells. Autophagy.

[43] Byun S (2017). A postprandial FGF19-SHP-LSD1 regulatory axis mediates epigenetic repression of hepatic autophagy. EMBO J..

[44] Wu B (2019). Epigenetic drug library screening identified an LSD1 inhibitor to target UTX-deficient cells for differentiation therapy. Signal Transduct. Target Ther..

[45] Barros Filho TE, Molina AE (2008). Analysis of the sensitivity and reproducibility of the Basso, Beattie, Bresnahan (BBB) scale in Wistar rats. Clinics (Sao Paulo).

[46] Li R (2017). Knockdown of ANRIL aggravates H_2_O_2_-induced injury in PC-12 cells by targeting microRNA-125a. Biomed. Pharmacother..

[47] Tian R, Shi R (2017). Dimercaprol is an acrolein scavenger that mitigates acrolein-mediated PC-12 cells toxicity and reduces acrolein in rat following spinal cord injury. J. Neurochem..

[48] Ma Z (2020). Rosmarinic acid exerts a neuroprotective effect on spinal cord injury by suppressing oxidative stress and inflammation via modulating the Nrf2/HO-1 and TLR4/NF-κB pathways. Toxicol. Appl. Pharm..

[49] Li H (2019). Mitochondrial transfer from bone marrow mesenchymal stem cells to motor neurons in spinal cord injury rats via gap junction. Theranostics.

[50] Wang G (2019). Schizandrin protects against OGD/R-induced neuronal injury by suppressing autophagy: involvement of the AMPK/mTOR pathway. Molecules.

